# Diarrhetic Shellfish Poisoning, Washington, USA, 2011

**DOI:** 10.3201/eid1908.121824

**Published:** 2013-08

**Authors:** Jennifer K. Lloyd, Jeffrey S. Duchin, Jerry Borchert, Harold Flores Quintana, Alison Robertson

**Affiliations:** Public Health—Seattle & King County, Seattle, Washington, USA (J.K. Lloyd, J.S. Duchin);; University of Washington, Seattle (J.S. Duchin);; Washington State Department of Health, Tumwater, Washington, USA (J. Borchert);; US Food and Drug Administration, Dauphin Island, Alabama, USA (H. Flores Quintana, A. Robertson)

**Keywords:** Diarrhetic shellfish poisoning, shellfish, toxins, DSP, dinophysistoxins, marine biotoxins, Dinophysis spp., Washington, United States, enteric infections

## Abstract

Diarrhetic shellfish poisoning is a gastrointestinal illness caused by consumption of bivalves contaminated with dinophysistoxins. We report an illness cluster in the United States in which toxins were confirmed in shellfish from a commercial harvest area, leading to product recall. Ongoing surveillance is needed to prevent similar illness outbreaks.

Diarrhetic shellfish poisoning (DSP) is an acute gastrointestinal illness caused by consumption of bivalve mollusks that have accumulated okadaic acid (OA) or related dinophysistoxins through filter feeding. DSP toxins are produced by several species of marine dinoflagellates from the genera *Dinophysis* and *Prorocentrum *(*1*–*4*). Symptoms of DSP include nausea, abdominal pain, vomiting, diarrhea, headache, chills, and fever (*5*). Onset occurs 0.5–4 hours after consumption of contaminated food, and symptoms last up to 72 hours; treatment is supportive. To date, no sequelae have been reported, but speculation has suggested that chronic exposure may increase risk for gastrointestinal cancers (*6**,**7*).

The earliest clinical reports of DSP were from the Netherlands in 1961; however, DSP toxins were structurally elucidated >15 years later in Japan (*2*,*8*,*9*). DSP illnesses have since been documented worldwide. In the United States, sporadic DSP-like illnesses have been recorded on the East Coast since 1980, coinciding with detection of toxin-producing dinoflagellates in shellfish beds (*2*,*4*). In 2002, shellfish beds in Chesapeake Bay, Virginia, were briefly closed because *Dinophysis* spp. dinoflagellates were detected, although hazardous DSP toxin levels were not detected and no illnesses were reported (*10*)*.* More recently, in Texas, harvest areas were closed for >1 month following a large *Dinophysis* bloom that contaminated oyster beds with OA in excess of the Food and Drug Administration (FDA) regulatory guidance level; no illnesses were reported (*11*,*12*)*.*

In the Pacific Northwest, *Dinophysis* spp. dinoflagellates, predominantly *D. acuminata,* have been observed for many years (*13*). During 2010, the Washington Department of Health (WDOH) and FDA Gulf Coast Seafood Laboratory (FDA-GCSL, Dauphin Island, AL) initiated a pilot program to gather baseline monitoring data on *Dinophysis* species abundance and associated DSP toxins in shellfish from 18 growing areas (Figure 1). During this pilot study, shellfish were collected for toxin analysis when *Dinophysis* light microscopy counts exceeded 3,000 cells/L for >2 consecutive weeks. During 2010, *Dinophysis* counts were reported above threshold at 15 sites, and >50 shellfish samples were analyzed for DSP toxins. All were below the FDA guidance level for total OA equivalents (free OA, DTX-1, DTX-2, and acyl esters) of 16 µg/100 g shellfish tissue. On the basis of these data, monitoring was scaled down to 5 field sites for ongoing cell monitoring and toxin analysis in 2011.

**Figure 1:**
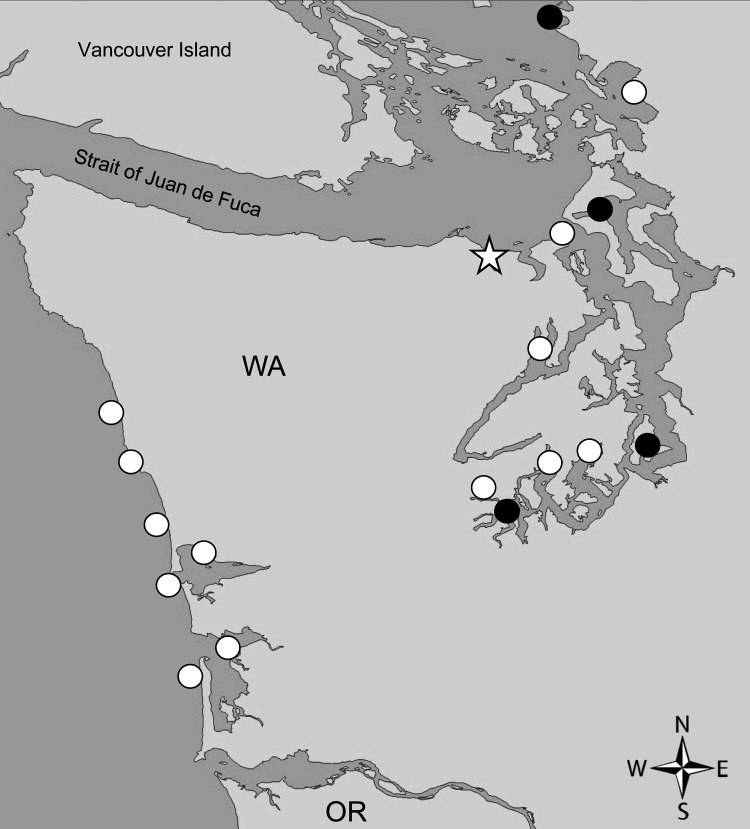
Diarrhetic shellfish poisoning toxin monitoring sites, Washington, USA, 2010–2011. Eighteen sites were monitored during the 2010 pilot study (open circles); 5 pilot sites were selected for continued monitoring during 2011 (solid circles). Sequim Bay State Park (star), the site implicated in the human illnesses described in this article, was among the sites monitored in 2011.

We describe a cluster of DSP illnesses that occurred in 2011 in which shellfish from the implicated harvest area exceeded the FDA regulatory guidance level for DSP toxins. This cluster resulted in closure of harvest areas and a recall of commercial shellfish product.

## The Study

In July 2011, Public Health–Seattle & King County received a report from WDOH of a family who experienced illness after consuming recreationally harvested mussels. Interviews were conducted to characterize the illness, determine the location of harvest, and describe preparation of the shellfish meal. Three family members, ages 2, 5, and 45 years, experienced symptoms beginning 4, 7, and 14 hours after consumption of cooked mussels, respectively. A fourth adult family member who consumed mussels did not become ill. Signs and symptoms included vomiting, diarrhea, body aches, fever, and chills; no neurologic symptoms were described. The average duration of vomiting and diarrhea was 3 and 52 hours, respectively. All ill persons recovered within 96 hours; no medications were taken, and medical care was not sought.

The family collected mussels from a public dock at Sequim Bay State Park, Sequim, Washington, on June 29, 2011. The mussels were stored in seawater until they were cooked 2 hours later. The mussels were boiled in water, wine, herbs, and butter for 10 minutes until the shells opened, then consumed immediately. The case-patients each consumed 8–15 mussels; the family member who did not become ill consumed 4 mussels.

**Figure 2:**
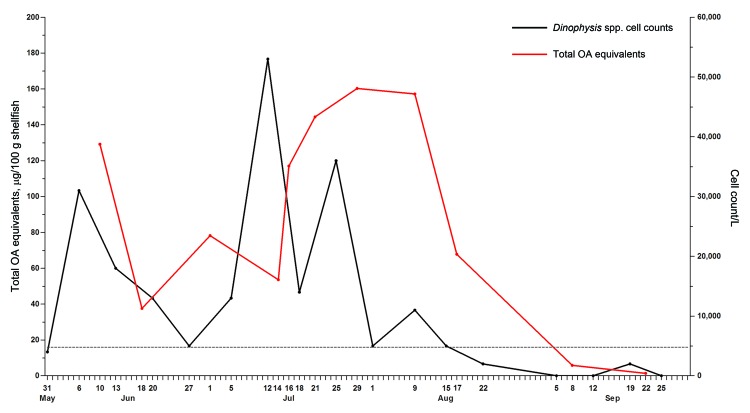
Timeline comparing blooms of *Dinophysis* spp. dinoflagellates and diarrhetic shellfish poisoning toxin levels detected in mussels collected during 2011 from Sequim Bay State Park, Sequim, Washington, USA. *Dinophysis* spp. cell counts per liter (black line) were determined by using light microscopy. Total okadaic acid (OA) equivalents (red line), in micrograms per 100 g shellfish tissue, were determined by using liquid chromatography mass spectrometry analysis (*14*). Dashed line indicates US Food and Drug Administration guidance level of 16 µg total OA equivalents per 100 g shellfish tissue. Dates shown are collection dates for each tested sample.

Although a meal remnant was not available, the dock from which the family collected mussels was a 2011 DSP monitoring site. Eleven composite samples of mussels (*Mytilus trossulus*) representing a minimum of 20 individuals had been collected before and after the collection date of the outbreak. Samples were received by FDA-GCSL on August 2, 2011, and hydrolyzed extracts were analyzed by using liquid chromatography tandem mass spectrometry (*14*). Analyses were performed under acidic chromatographic conditions and performed in negative-ion mode by multiple-reaction monitoring. Matrix dilution experiments were performed to ensure that sample matrix did not suppress or enhance ionization during the analyses. Total OA equivalents (free OA, DTX-1, DTX-2, and acyl esters of OA, DTX-1, and DTX-2) were quantified by external standard calibration with OA. Certified reference standards (OA, DTX-1, and DTX-2) from the National Research Council (Halifax, Nova Scotia, Canada) were used for verification of retention time and equimolar response. Nine mussel samples exhibited toxin levels above the FDA guidance threshold, ranging from 37.6 µg–160.3 µg total OA equivalents per 100 g. In all cases, DTX-1 was identified as the principal DSP toxin. These elevated DSP toxin levels followed observed *Dinophysis* blooms (Figure 2).

On the basis of the analytical results, a recall was initiated for clams and oysters harvested after August 1, 2011, from the commercial growing area adjacent to Sequim Bay State Park; no mussels were harvested commercially. The park and commercial site remained closed until 2 consecutive shellfish samples collected 7–10 days apart demonstrated total DSP toxin levels <16 µg/100 g. Clams and oysters were cleared for commercial harvest on September 2, 2011; recreational mussel harvest was allowed starting in late October. During these closures, WDOH issued a press release and posted information online describing the dangers of DSP. Warning signs instructing visitors not to collect shellfish for consumption were posted at affected beaches. Similar warnings were issued on the WDOH biotoxin hotline and online maps of shellfish harvest areas (www.doh.wa.gov/shellfishsafety.htm). Surveillance for DSP illnesses is ongoing; at the time this article was written, no additional reports had been received.

## Conclusion

We describe a cluster of DSP illnesses in the US Pacific Northwest with confirmation of DSP toxins in shellfish from the implicated harvest area. Mussels contained levels of DSP toxin 2–10 times the guidance level, resulting in closure of recreational and commercial areas. Coincidentally, roughly 60 DSP illnesses occurred in July–August 2011 in British Columbia, Canada, and were traced to Pacific Coast mussels (*15*). 

Although *Dinophysis* spp. dinoflagellates have been found in Pacific Coast waters for many years, illnesses consistent with DSP have not been reported. Research is ongoing to determine why elevated toxin levels are being observed in the region now. Underreporting of DSP is possible because of the nonspecific nature of the illness. To detect cases of DSP and other shellfish-related illnesses, clinicians should inquire about shellfish consumption preceding onset of symptoms. Ill patients with a history of shellfish consumption should be reported to public health authorities immediately to prevent further illnesses.

Liquid chromatography tandem mass spectrometry is a preferred method for FDA regulatory testing of marine biotoxins because it provides quantification and unambiguous identification of toxin congeners. WDOH is now equipped with this instrumentation and received FDA training in chemical methods for DSP toxins, which allows local monitoring of >40 shellfish growing areas on a regular basis. Such surveillance efforts are critical for early warning of toxicity and prompt response to this emerging public health issue. Relationships with and frequent communication between public health and shellfish program staff regarding human illness, increases in bloom frequency, and hazardous levels of DSP toxins in locally harvested shellfish will facilitate preventions of additional illnesses.
